# Effectiveness of Herbal Medicines with Anti-Inflammatory, Antimicrobial, and Antioxidant Properties in Improving Oral Health and Treating Gingivitis and Periodontitis: A Systematic Review

**DOI:** 10.3390/nu17050762

**Published:** 2025-02-21

**Authors:** Giuseppina Malcangi, Angelo Michele Inchingolo, Lucia Casamassima, Irma Trilli, Laura Ferrante, Francesco Inchingolo, Andrea Palermo, Alessio Danilo Inchingolo, Gianna Dipalma

**Affiliations:** 1 Department of Interdisciplinary Medicine, University of Bari “Aldo Moro”, 70121 Bari, Italy; giuseppinamalcangi@libero.it (G.M.); lucia.casamassima@uniba.it (L.C.); irmatrilli@hotmail.com (I.T.); lauraferrante79@virgilio.it (L.F.); alessiodanilo.inchingolo@uniba.it (A.D.I.); giannadipalma@uniba.it (G.D.); 2 Department of Experimental Medicine, University of Salento, 73100 Lecce, Italy; andreapalermo@unisalento.it

**Keywords:** herbal medicine, plant extracts, essential oils, phytochemicals, antibacterial activity, gingivitis, periodontitis, anti-inflammatory activity, dentistry oral health, periodontal therapy

## Abstract

Objectives: This systematic review investigates the effectiveness of natural extracts with anti-inflammatory properties for improving oral health, particularly in managing gingivitis and periodontal disease (PD). With PD being a major global health issue, exacerbated by microbial dysbiosis and oxidative stress, the integration of phytochemicals and herbal formulations into periodontal therapy offers a promising avenue for adjunctive treatments. Methods: A systematic review was conducted following PRISMA guidelines and registered under the International Prospective Register of Systematic Reviews (ID: 641944). Databases, including PubMed, Scopus, and Web of Science, were searched between 18–24 December 2024, using Boolean keywords combining terms such as “herbal medicine”, “plant extracts”, “anti-inflammatory”, and “periodontal therapy”. Studies involving animal models, in vitro data, or non-peer-reviewed articles were excluded. Results: Seventeen studies met inclusion criteria. Polyherbal formulations and single-component extracts (e.g., *Camellia sinensis*, *Punica granatum*, *Zingiber officinale*, and *Rosmarinus officinalis*) demonstrated comparable efficacy to conventional agents like chlorhexidine (CHX). Polyherbal rinses, *camellia sinensis* gels, and extracts like *Punica granatum* reduced inflammation, improved gingival health, and showed antimicrobial properties, offering effective natural alternatives. Conclusions: Natural products, including single extracts and polyherbal formulations, provide effective and safe alternatives for managing gingivitis and PD. Their anti-inflammatory, antimicrobial, and antioxidant properties support their adjunctive role alongside with scaling and root planning therapy (SRP) in periodontal therapy. However, further large-scale, long-term studies are needed to standardize formulations and establish optimal protocols.

## 1. Introduction

Dental caries (DC) and periodontal disease (PD) continue to be the most prevalent public health problems in the world. Although recent surveys show a decrease in the incidence and severity of carious lesions, PD remains the sixth most common disease worldwide, with 10% of the population affected by severe PD [1,2,3,4,5,6,7].

PD is a degenerative inflammatory disease of the periodontium caused by an altered oral microbiota associated with a reduced immune response of the body [8,9,10].

It involves the production of pro-inflammatory cytokines, particularly IL-1β, PGE2 (linked to bone resorption and PD severity), and MMPs (especially MMP-8 and MMP-9), which degrade collagen and contribute to periodontal tissue destruction [11,12,13,14,15,16,17]. Other metabolic, neurodegenerative, autoimmune, and oncological diseases are often associated with PD [18,19,20]. The potential link between PD and systemic diseases has led to research into new treatment options and a greater emphasis on periodontal treatment and control, as well as early diagnosis [21,22].

PD is diagnosed by assessing the plaque index (PI), bleeding on probing (BOP), the depth of the periodontal pocket, and the extent of gingival recession, and by testing for biomarkers (MMP-8 and MMP-9, PGE2 and IL-1β) that are detectable in the crevicular fluid [23,24,25,26].

To assess the therapeutic efficacy of natural products in periodontal health, various clinical parameters and biomarkers are commonly evaluated. The primary indicators include the Plaque Index (PI) and Bleeding on Probing (BOP), which provide insights into plaque accumulation and gingival inflammation. Additionally, reductions in periodontal pocket depth (PPD) and gingival recession (GR) are used to measure improvements in periodontal structure. At a molecular level, changes in biomarkers such as matrix metalloproteinases (MMP-8 and MMP-9), prostaglandin E2 (PGE2), and interleukin-1β (IL-1β) in gingival crevicular fluid (GCF) serve as objective indicators of inflammation modulation. In several clinical studies, the efficacy of plant-derived mouthwashes and gels has been confirmed through these parameters, showing significant reductions in inflammatory markers and improvements in periodontal health compared to conventional treatments. PD management typically involves specialist visits, home hygiene, CHX mouthrinses, and antibiotics like metronidazole. However, prolonged CHX use may cause mucosal and dental staining, taste loss, irritation, and antibiotic resistance [27,28,29,30].

In recent years, the scientific community has developed an interest in the use of natural products, rich in bioactive substances with antimicrobial, bacteriostatic, immunomodulatory, and restorative properties, known since ancient times, capable of slowing down, controlling, or preventing PD [31,32,33]. About 35,000 medicinal plants are known, but only 20% are analyzed phytochemically, and of these, only 10% are biologically screened to ensure quality and non-toxicity [34,35,36,37,38,39]. Extracts, concentrated forms of phytochemicals known as nutraceuticals, have received particular attention [40,41].

There are many ways to extract substances from plants, from long boiling to cold infusion, or by using alcohol, vinegar, hot water, or other solvents such as acetone and chloroform [42,43,44].

Nutraceuticals are bioactive substances that can be used as main therapeutic agents or as a basis for the synthesis of other drugs (e.g., morphine from opium, extracted from *Papaver somniferum*) [45,46,47].

**Figure 1:**
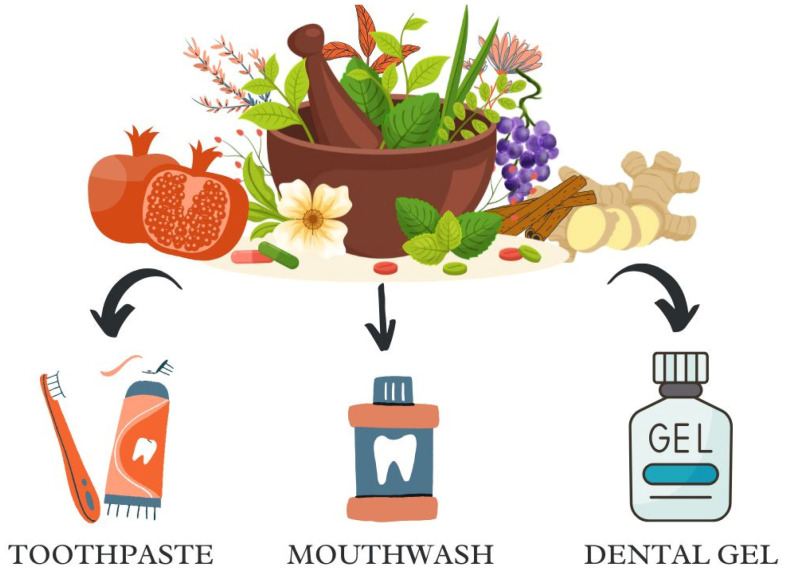
This image illustrates using extracts from natural ingredients (such as *punica granatum*, *flowers, zingiber, grapes*, and *cinnamon*) in oral care products. It shows the transformation of these natural components into three different types of dental products: toothpaste, mouthwash, and dental gel, highlighting a herbal approach to dental hygiene.

Many natural substances have been the subject of research for the treatment of oral cavity inflammation. The use of herbal extracts alone or in combination with other natural herbal extracts (triphala, sage, green tea, tulsi patra, jyestiamadh, neem, clove oil, pudina, ajwain, ginger, calendula, aloe vera, propolis, mangosteen extract complex, elderberry) in the form of mouthwashes, gels, and toothpastes (Figure 1) have been scientifically tested for various oral health problems: gingivitis, bleeding, bad breath, soft-tissue ulcers, infections, and prevention of the onset of DC [8,48,49,50,51,52,53]. These benefits are largely attributed to bioactive compounds such as polyphenols, flavonoids, tannins, and terpenoids, which exhibit anti-inflammatory, antimicrobial, and antioxidant properties. The active ingredients of phytotherapeutic products have the advantage that they have no side effects and are not harmful even in the medium and long term. In addition, all natural herbal mouthwashes do not contain sugar and/or alcohol, which are present in the vast majority of over-the-counter oral hygiene products, and can be a good substrate for the development of microorganisms [54,55,56,57]. Herbs from the *Lythraceae* family have high phytotherapeutic properties, including *Punica granatum* (pomegranate) and *Lawsonia inermis* (henna), whose extracts have shown remarkable anti-inflammatory properties [58,59,60,61]. Both extracts were used as mouthwashes, and the reduction in gingival and periodontal inflammatory status was correlated to the decrease in salivary enzyme activity of aspartate aminotransferase (AST), alanine aminotransferase (ALT), and lactate dehydrogenase (LDH), with a greater reduction with *Punica granatum* mouthwash [34,62,63]. Pomegranate extract owes its beneficial effects to the presence of punicalin, phenols, flavonoids, and triterpenoids, which are mainly found in the rind. The bioactive components reduce inflammatory mediators, stimulate collagen production, and angiogenesis and reduce bacterial proliferation. *Lawsonia inermis* owes its antibacterial properties to the presence of 2-hydroxynaphthoquinone, saponins, and xanthones. *Punica granatum* extract, rich in punicalin, phenols, flavonoids, and triterpenoids, reduces inflammation, promotes collagen production, and inhibits bacterial growth. Similarly, other bioactive compounds such as tannins, terpenoids, and saponins found in medicinal plants contribute to oral health improvement by modulating inflammatory responses and reducing microbial adhesion. *Lawsonia inermis*’ antibacterial effects stem from 2-hydroxynaphthoquinone, saponins, and xanthones [64,65,66,67]. Polyherbal mouthwashes based on extracts of rosemary (*Rosmarinus officinalis Lamiaceae*), *Zingiber officinale*, and marigold (*Calendula officinalis*) have been produced which have shown efficacy in the treatment and prophylaxis of gingivitis comparable to that of CHX mouthwash without adverse effects [68,69,70,71,72]. Rosemary extract is a spice. It belongs to the *Lamiaceae* family and is also used in medicine for its many therapeutic properties and high antioxidant and antibacterial activity, thanks to the presence of active compounds: phenolic diterpenes (carnosic acid, but also carnosol, rosmanol and epi- and isorosmanol), flavonoids, and terpenoids. The healing of oral ulcers and gingivitis has been improved by its use in dentistry. *Rosmarinus* extract, a Lamiaceae spice with strong antioxidant and antibacterial properties, aids oral ulcer and gingivitis healing due to its phenolic diterpenes, flavonoids, and terpenoids. [73,74,75,76,77,78,79,80,81]. Ginger, *Zingiber officinale Roscoe*, a culinary spice, is widely used in phytotherapy. It has antimicrobial, antifungal, and pharmaceutical properties due to the presence of gingerol *zingiber* [82,83,84,85]. The mechanisms by which ginger *zingiber* inhibits the growth of oral bacteria that contribute to PD are still poorly understood [34,73,74,75,76,86,87]. However, it was found that ginger reduces PR synthesis and that two highly alkylated extracts in particular inhibit three anaerobic Gram-negative bacteria that cause PD: *Porphyromonas gingivalis* ATCC (American Type Culture Collection) 53978, *Porphyromonas endodontalis* ATCC 35406, and *Prevotella intermedia* ATCC 25611 at low doses (6–30 µg/mL). It involves the production of pro-inflammatory cytokines, particularly IL-1β, PGE2 (linked to bone resorption and PD severity), and MMPs (especially MMP-8 and MMP-9), which degrade collagen and contribute to periodontal tissue destruction [77,78,79,80,81,88,89,90,91,92,93,94,95]. The triterpenoids in the CO_2_ (carbon dioxide) extract of calendula flowers, including faradiol monoester, demonstrated predominant anti-inflammatory activity [96,97,98,99,100,101,102,103,104,105,106]. A 2% gel based on *Calendula officinalis* flowers is also used as an individual treatment for inflammation of the oral mucosa and oropharyngeal mucositis caused by radiotherapy and chemotherapy [62,77,107,108,109,110,111,112]. The combined extract of *propolis* and *Propolis* and *Mangosteen Extract Complex* (*PMEC*) was used to treat patients with advanced gingivitis and PD [113,114,115,116,117,118,119,120,121,122]. The results showed a strong therapeutic response with reduction of all biomarkers present in GCF (IL-1β: 57.9%, PGE2: 51.1%, MMP-8: 46.3%, and MMP-9: 54.4%), including macrophages, gingival fibroblasts, and neutrophils, resulting in improved osteogenesis, decreased osteoclastic activity, and reduced alveolar bone resorption. However, the use of PMEC found little short-term clinical improvement due to its inability to prevent direct invasion of the gingival tissue by periodontal pathogens. It reduced GCF biomarkers and improved osteogenesis, but *PMEC* had limited short-term clinical benefits due to persistent pathogen invasion [123,124,125]. Mouthwashes with natural herbs (*neem*, *tulsi, pudina, clove* oil, *camellia sinensis* green tea, *aloe vera*, *ajwain, triphala* and many others) have been formulated to treat bleeding gums, bad breath, and mouth ulcers, and to prevent tooth decay. Herbal and essential oil mouthwashes have been used [49,126,127,128,129]. This study provides a novel perspective by systematically evaluating not only the anti-inflammatory properties of herbal formulations in periodontal therapy but also their antimicrobial and antioxidant effects, highlighting their potential as comprehensive adjuncts to conventional treatments.

## 2. Materials and Methods

### 2.1. Search Processing

The current systematic review followed the PRISMA (Preferred Reporting Items for Systematic reviews and Meta-Analyses) guidelines and International Prospective Register of Systematic Review Registry procedures (full ID: 641944). The databases PubMed, Web of Science, and Scopus were examined from 18 December 2024 to 24 December 2024 to search articles of the last 10 years (Table S1). The search strategy was created by combining terms relevant to the study’s purpose. The following Boolean keyword search was applied: (“herbal medicine” OR “plant extracts” OR “essential oils” OR “phytochemicals”) AND (“anti-inflammatory” OR “anti-inflammatory activity”) AND (“dentistry” OR “oral health” OR “periodontal therapy” OR “gingivitis” OR “periodontitis”)).

### 2.2. Inclusion and Exclusion Criteria

The reviewers worked in groups to assess all relevant studies that evaluated or compared the effectiveness of natural extracts with anti-inflammatory properties in improving oral health and treating gingivitis and PD, following the inclusion criteria below:

- Studies with open access written in English.
- Studies that did the research “in vivo” or in “humans”.
- Case-control studies, cohort studies, randomized controlled trials (RCTs).
- Studies that were published in the last 10 years.
- Articles related to adult studies.

Studies that fulfilled at least one of the following exclusion criteria were excluded: reviews, case reports and series, letters to the authors, animal models, and in vitro studies.

### 2.3. PICo Question

The PICo format is a framework used in qualitative research to structure clinical research questions. The PICo addressed the question: “*What is the effectiveness of herbal medicine with anti-inflammatory properties in improving oral health or treating gingivitis and periodontitis in adult patients?*”

I. P (Population): Adult patients with gingivitis or periodontitis.
II. I (Phenomenon of Interest): Effectiveness of herbal medicine with anti-inflammatory properties.
III. Co (Context): It applies to improving oral health or treating gingivitis and periodontitis.

### 2.4. Data Processing

Four independent reviewers (L.C., L.F., I.T., and G.M.) assessed the included studies’ quality using criteria such as selection criteria, methods of outcome evaluation, and data analysis. This enhanced ‘risk of bias’ tool additionally provides quality standards for selection, performance, detection, reporting, and other biases. All differences were settled through conversation or collaboration with other researchers (G.D., A.D.M., and A.M.I.). The reviewers screened the records according to the inclusion and exclusion criteria. The 301 selected articles were downloaded into “Zotero 6.0.36” for organization and analysis.

## 3. Results

### 3.1. Selection and Characteristics of the Study

A total of 301 publications were identified through online databases: PubMed (*n* = 43), Scopus (*n* = 185), and Web of Science (*n* = 73). No additional studies were identified through manual research. After removing 2 duplicate records, 299 studies remained and were screened by title and abstract.

**Figure 2:**
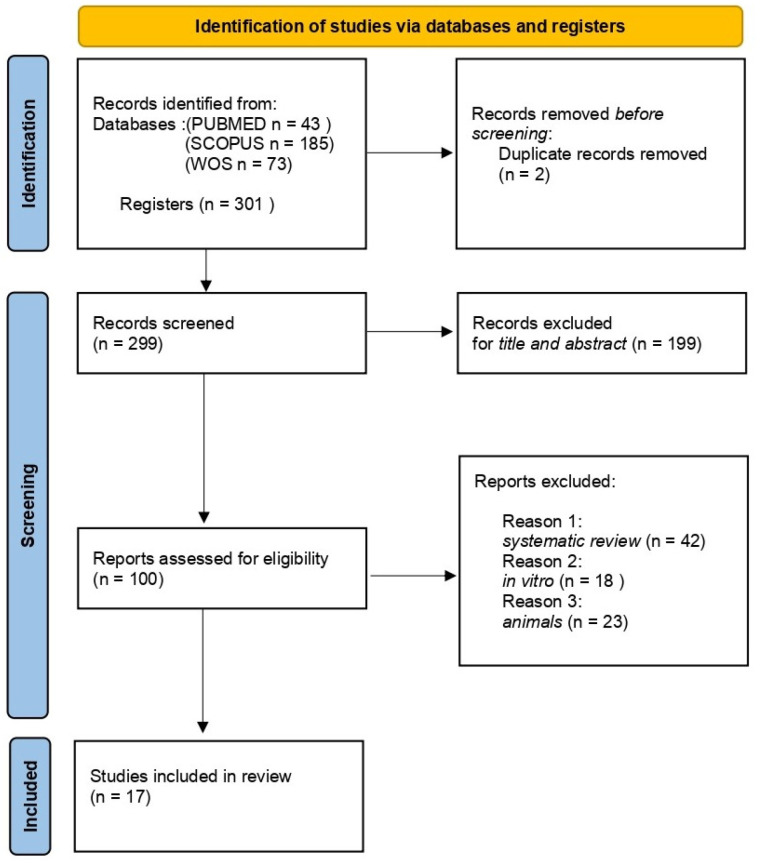
PRISMA flowchart.

Following a detailed eligibility assessment of the 100 reports, 83 were excluded for not meeting the criteria, leaving 17 studies for qualitative analysis. The selection process and summary of included records are illustrated in Figure 2.

### 3.2. Key Results and Summary

The primary outcomes considered for each study included plaque index (PI), gingival index (GI), bleeding index (BI), bleeding on probing percentage (BOP%), and clinical attachment level (CAL).

A summary of key findings from selected studies is provided below, while the full dataset is available in Table S2:

- *Camellia sinensis*-based dentifrice significantly reduced gingival inflammation and increased antioxidant activity in gingival crevicular fluid.
- Polyherbal mouthwash (*Zingiber* officinale, *Rosmarinus* officinalis, *Calendula* officinalis) showed comparable efficacy to chlorhexidine (CHX) in reducing gingivitis.
- Locally delivered *Camellia sinensis* gel provided clinical benefits in periodontitis management but had no significant long-term superiority over SRP alone.
- *Grape* seed extract gel (2%) improved GI and PI but showed no significant differences in probing depth (PoD).
- Cinnamon extract as an ultrasonic coolant effectively reduces bacterial contamination in aerosols, offering a cost-effective alternative to CHX.
- *Rhizoma chuanxiong* and *Rhizoma imperatae* toothpaste improved GI, BI, and BOP% significantly after 12 weeks.
- *Podila* extract toothpaste showed sustained improvement in PI, GI, BI, and BOP%, with no significant adverse effects.
- Sage-containing mouthwash had no superior effect over placebo in reducing Sulcus Bleeding Index (SBI) and PI.
- *Punica granatum* and *Lawsonia inermis* mouthwashes significantly reduced salivary inflammation markers, with *Punica granatum* showing superior efficacy.

These results emphasize the potential of herbal medicine in oral health care, particularly in reducing gingival inflammation, plaque accumulation, and bacterial load, with some extracts offering comparable efficacy to conventional treatments like CHX.

### 3.3. Quality Assessment and Risk of Bias of Included Articles

The quality assessment and risk of bias evaluation using the “Risk of Bias in Non-Randomized Studies of Interventions” (ROBINS-I) tool included 17 studies, as reported in Table S3. This evaluation assessed six domains (D1–D6) across multiple studies. Bias due to confounding (D1): all studies effectively controlled for potential confounding factors by implementing strategies such as randomization, and clearly defined inclusion and exclusion criteria. This ensured that the results were reliable and attributable to the interventions being tested. Bias arising from measurement of the exposure (D2): the measurement process was consistently standardized across the studies, resulting in a low risk of bias in this domain. Bias in the selection of participants (D3): most studies maintained a low risk by employing proper selection processes. However, a few studies showed some concerns, suggesting minor issues in participant recruitment or allocation. Bias due to post-exposure interventions (D4): all studies effectively standardized post-exposure interventions, resulting in a consistently low risk of bias in this domain. Bias due to missing data (D5): some studies were flagged for missing data, leading to some concerns. This highlights areas where dropouts or incomplete data handling could potentially influence the outcomes. Bias arising from the measurement of the outcome (D6): most studies demonstrated low risk in this domain, utilizing validated and objective outcome measures. However, a few instances of some concerns were noted.

The majority of the studies demonstrated a low overall risk of bias, reflecting methodological rigor and reliable findings. Nonetheless, a few areas with some concerns, particularly regarding missing data and participant selection, highlight opportunities for improved reporting and data handling in future research.

## 4. Discussion

PD and gingivitis represent chronic inflammatory conditions primarily caused by microbial dysbiosis and exacerbated by oxidative stress [130,131,132,133,134]. Conventional treatments, including SRP, are often augmented with chemotherapeutics or antioxidants to enhance outcomes. The integration of natural and plant-based products as adjunctive therapies has gained substantial attention due to their antimicrobial, antioxidant, and anti-inflammatory properties. This review synthesizes findings from 17 studies investigating the efficacy of various natural compounds and formulations in managing periodontal conditions [135,136,137,138,139,140,141,142,143,144,145,146]. To provide a clearer overview of the bioactive components responsible for the beneficial effects of natural products in oral health, Table S4 summarizes the key plant species included in this review along with their main active compounds and associated therapeutic properties. Summary of plant-based products with their effects on oral cavity is represented in Table S5.

### 4.1. Camellia sinensis-Based Interventions

T. S. Hrishi et al. (2016) explored the adjunctive role of *Camellia sinensis*-based dentifrice in treating mild-to-moderate PD. Their randomized clinical trial with 30 patients compared outcomes between a test group using *Camellia sinensis* dentifrice and a control group using commercial toothpaste [147]. The clinical parameters GI, PI, probing PoD, and CAL—along with antioxidant markers in GCF, were evaluated over four weeks. While both groups improved due to SRP, the *Camellia sinensis* group showed significantly greater reductions in gingival inflammation and enhanced antioxidant activity. This suggests the potential of *Camellia sinensis* as a beneficial adjunct, though larger-scale studies are warranted [147,148,149,150,151,152]. Similarly, Kanyawat Rattanasuwan et al. (2016) investigated a locally delivered *Camellia sinensis* gel in chronic PD. Forty-two participants underwent SRP, followed by application of either *Camellia sinensis* gel or placebo. While both groups showed significant clinical improvements, the *Camellia sinensis* gel demonstrated superior anti-inflammatory effects at three months, though its impact on long-term periodontal outcomes was inconclusive. The study emphasized the need for larger trials to determine optimal protocols and long-term efficacy [153].

### 4.2. Polyherbal Mouthwashes

Saman Mahyari et al. (2016) evaluated a polyherbal mouthwash containing extracts of *Zingiber* officinale, *Rosmarinus* officinalis, and *Calendula officinalis* in gingivitis management [68]. Sixty participants were divided into polyherbal, CHX, and placebo groups, with assessments over 14 days. Both the polyherbal and CHX mouthwashes significantly improved clinical indices, demonstrating comparable efficacy. Polyherbal formulation had no adverse effects, suggesting it as a viable alternative to synthetic products [51,68,154,155,156]. In 2019, Scilla Sparabombe et al. examined another polyherbal mouthwash containing *propolis*, *plantago lanceolata, salvia officinalis* and essential oils. Over three months, the mouthwash significantly reduced inflammation (FMBS) and plaque accumulation (FMPS), particularly in moderate-to-severe PD cases, without adverse effects. The findings underscore its potential as a safe and effective alternative to conventional CHX mouthwash [157].

### 4.3. Herbal Toothpastes

He (2019) conducted a double-blind trial assessing toothpaste containing *Rhizoma Chuanxiong* and *Rhizoma Imperatae* extracts. Over 12 weeks, 120 participants showed significant reductions in GI, BI, and BOP in the test group compared to controls. The herbal toothpaste demonstrated no adverse effects, highlighting its efficacy and safety for managing gingivitis [158]. Li Cheng et al. (2019) tested *Pudilan* extract-containing toothpaste, composed of flavonoids, alkaloids, and organic acids, over a 12-week period. Significant improvements in PI, GI, BI, and BOP were observed in the experimental group compared to placebo. The gradual improvement over time suggests potential long-term benefits of *Pudilan* extract in chronic gingivitis management [159]. In 2020, Zaira F. Kharaeva explored a toothpaste combining sodium monofluorophosphate, xylitol, and Swiss herbal extracts, including Chamomilla recutita and Salvia officinalis. Results indicated significant reductions in inflammation, plaque presence, and early signs of PD compared to controls [160]. The study highlighted the synergistic effects of chemical and plant-derived antibacterials in managing PD [160,161,162,163,164].

### 4.4. Alternative Herbal Agents in Gingivitis and Periodontitis

Mohammad Rayyan et al. (2018) assessed a 2% mucoadhesive *Grape* Seed Extract (*GSE*) gel in chronic PD [165]. Over six months, the *GSE* group showed significant reductions in GI and PI compared to controls, though PoD improvements were limited. The authors suggested that higher *GSE* concentrations might yield better results [165,166,167,168]. Alefiya Shabbir Mamajiwala et al. (2018) compared CHX and cinnamon extract as ultrasonic coolant agents during scaling. Both significantly reduced bacterial contamination in aerosols, with cinnamon extract demonstrating comparable efficacy without side effects like staining. This positions cinnamon extract as a cost-effective alternative for low-resource settings [169]. Eltay et al. (2021) studied a solution containing 5% *Punica granatum* extract as a supplement to non-surgical therapy for chronic gingivitis. Over four weeks, *Punica granatum* significantly reduced PI, GI, and IL-1β levels compared to placebo, demonstrating potent anti-inflammatory and antimicrobial properties without adverse effects. These findings support its potential for safe and natural periodontal therapy [170]. Takkella et al. (2021) compared mouthwashes containing *Punica granatum* and *Lawsonia inermis*. Both reduced salivary enzymes linked to inflammation and tissue damage, with *punica granatum* showing superior efficacy due to its bioactive compounds promoting collagen synthesis and reducing bacterial proliferation. The study recommended herbal mouthwashes as safe alternatives to alcohol-based formulations [58].

### 4.5. Propolis and Other Natural Extracts

Jaber Yaghini et al. (2019) compared *aloe vera camellia sinensis*, matrica, and CHX mouthwashes in plaque-induced gingivitis. *Aloe vera camellia sinensis* was as effective as CHX in reducing plaque and gingival inflammation while causing less dental staining. The findings highlight its promise as an alternative to conventional chemical mouthwashes [171]. Hendrik Jünger et al. (2020) investigated a sage-based mouthwash in elderly patients receiving supportive periodontal care. Although improvements in gingival inflammation were observed, they were not statistically significant compared to placebo. The study suggested that the antimicrobial properties of alcohol and mint oil in the placebo might have confounded results. Future research without alcohol-based controls was recommended [172]. Jung et al. (2024) evaluated a *PMEC* compound for moderate-to-severe gingivitis. Over eight weeks, *PMEC* significantly reduced inflammatory biomarkers in GCF, including IL-1β and PGE2, though clinical improvements in GI and BOP were modest. The authors demonstrated the significant reductions in gingival crevicular fluid biomarkers, by explaining the mechanism of inhibition that the *PMEC* complex explicates on the expression of pro-inflammatory cytokines (IL-1β and PGE2) and metalloproteases (MMP-8 and MMP-9) in periodontal tissue cells infected with oral bacteria, including neutrophils, macrophages, and gingival fibroblasts; such inhibition, in fact, reduces the process of osteoclastogenesis and conversely promotes the osteogenetic one, thereby reducing alveolar bone destruction. However, *PMEC* does not prevent the direct invasion of periodontal pathogens into gingival tissue cells or the subsequent release of lipopolysaccharide (LPS) and exotoxins into the gingival capillaries, which is why the study still shows a discrepancy between results of clinical parameters in *PMEC*-treated and placebo-treated groups. The study highlighted *PMEC*’s potential anti-inflammatory effects and called for longer-term trials [173].

### 4.6. Innovative Approaches and Phytocompounds

Aslan Kh. Sheregov et al. (2021) assessed *standardized fermented papaya* gel (*SFPG*) in moderate chronic PD associated with orthodontic treatment. *SFPG* significantly reduced inflammation and cytokine levels, emphasizing its antioxidant and antimicrobial properties as a topical adjunct to conventional therapy [174]. Young Park et al. (2021) evaluated *PMEC* capsules in early gingivitis and PD. Over two months, *PMEC* reduced GI and IL-6 levels in GCF, indicating anti-inflammatory benefits. However, improvements in clinical parameters like periodontal depth were not statistically significant. The study highlighted the potential for systemic phytocompounds in periodontal therapy [175]. Yu Rin Kim et al. (2022) examined a Sambucus williamsii var. coreana-based mouthwash. Significant reductions in periodontal indices and pathogenic bacteria were observed, showcasing the mouthwash’s immediate and progressive antibacterial effects. The study underscored the potential of natural alternatives to prevent and treat dental diseases [176]. The reviewed studies collectively emphasize the potential of natural and herbal compounds in managing gingivitis and PD. Products like *Camellia sinensis*, *Polyherbal mouthwashes, GSE* and *Punica granatum* demonstrate significant anti-inflammatory, antioxidant, and antimicrobial properties. While many natural agents show promise as safe alternatives to conventional treatments like CHX, limitations include small sample sizes, short follow-up periods, and variations in study design. Further large-scale, long-term studies are necessary to validate their clinical efficacy and establish standardized protocols for integration into periodontal care. Nonetheless, these findings pave the way for innovative, plant-based solutions that address oral health challenges while minimizing side effects associated with synthetic agents [177,178,179].

### 4.7. Clinical Implications of Structure–Activity Correlations

The therapeutic efficacy of plant-derived compounds in managing periodontal diseases is largely determined by their chemical structure, which governs their bioavailability and biological activity. Understanding these structure–activity relationships can help elucidate the mechanisms through which natural compounds exert their antimicrobial, anti-inflammatory, and antioxidant effects in periodontal therapy.

*Polyphenols* (e.g., catechins from *Camellia sinensis*): the presence of multiple hydroxyl groups enables metal ion chelation and free radical neutralization, reducing oxidative stress in periodontal tissues. Additionally, catechins interact with bacterial membrane proteins, inhibiting bacterial adhesion and biofilm formation.

Ellagitannins (e.g., punicalagins from *Punica granatum*): These tannins act as potent MMP inhibitors, preventing the degradation of collagen and extracellular matrix components. They also suppress IL-1β and TNF-α production, thereby modulating inflammatory responses and limiting tissue destruction.

Terpenoids (e.g., carnosic acid from *Rosmarinus officinalis*): Due to their lipophilic nature, terpenoids integrate into bacterial membranes, increasing their permeability and leading to cell lysis. This mechanism underlies their broad-spectrum antimicrobial properties.

Flavonoids (e.g., quercetin from *Cinnamomum verum* and *Zingiber officinale*): Flavonoids regulate NF-κB signaling pathways, thereby reducing the transcription of pro-inflammatory cytokines such as IL-1β, TNF-α, and PGE2. This anti-inflammatory action helps control gingival inflammation and periodontal tissue damage.

Polysaccharides (e.g., acemannan from *Aloe vera*): These compounds stimulate fibroblast proliferation and enhance collagen synthesis, promoting tissue regeneration and wound healing. Additionally, acemannan exhibits immune-modulatory effects, reducing inflammatory mediator release [160,161,162,163,164].

These structure–activity correlations highlight the pharmacological potential of phytochemicals in periodontal therapy. By targeting key molecular pathways involved in oxidative stress, bacterial proliferation, and inflammation, plant-based compounds serve as valuable adjuncts to conventional periodontal treatments. However, further studies are needed to standardize formulations, optimize bioavailability, and confirm long-term clinical efficacy.

## 5. Conclusions

This systematic review collects various clinical studies that explore the use of natural remedies and supplements as complementary treatments for PD, including gingivitis and PD. The conclusions of this systematic review are as follows:

- Potential of Plant-Based Remedies: Natural extracts like *Camellia sinensis*, cinnamon, *aloe vera* and *rosmarinus* show promise in reducing inflammation, plaque, and bleeding in PD.
- Effectiveness Compared to Conventional Treatments: While these remedies offer benefits, they often do not surpass conventional options like CHX or antibiotic gels.
- Safer Alternative with Fewer Side Effects: Some plant-based treatments, such as cinnamon, have performed similarly to CHX but without side effects like dental staining.
- Temporary and Variable Effects: The benefits of these natural remedies are often short-lived, and results vary across studies, highlighting the need for standardization.

Overall, this review supports the idea that some natural extracts could be a valuable aid in the management of PD, but further investigations, with larger sample sizes and longer follow-ups, are necessary to determine their definitive role as first-line treatments or as supplements to existing therapies.

## Supplementary Materials

The following supporting information can be downloaded at: https://www.mdpi.com/article/10.3390/nu17050762/s1.

## Abbreviations

ALT	Alanine Aminotransferase
AST	Aspartate Aminotransferase
ATCC	American Type Culture Collection
BI	Bleeding Index
BOP	Bleeding on Probing
CAL	Clinical Attachment Level
CHX	Chlorhexidine
CO2	Carbon Dioxide
CTP	Control Toothpaste
DC	Dental Caries
DW	Distilled Water
FMBS	Full Mouth Bleeding Score
FMPS	Full Mouth Plaque Score
GCF	Gingival Crevicular Fluid
GBI	Gingival Bleeding Index
GI	Gingival Index
GSE	Grape Seed Extract
IL	Interleukin
IL-6	Interleukin-6
LDH	Lactate Dehydrogenase
MGI	Modified Gingival Index
MMPs	Metalloproteinases
MQH	Modified Quigley-Hein Index
PD	Periodontal Disease
PoD	Pocket Depth
PI	Plaque Index
*PMEC*	*Propolis-Mangosteen* Extract Complex
PPD	Probing Pocket Depth
PR	Prostaglandin
PROMs	Patient-Reported Outcomes Measures
RCT	Randomized Controlled Trial
SBI	Sulcus Bleeding Index
SFPG	Fermented Papaya Gel
SRP	Scaling and Root Planing
