# Supplementary material for: Effectiveness of Herbal Medicines with Anti-Inflammatory, Antimicrobial, and Antioxidant Properties in Improving Oral Health and Treating Gingivitis and Periodontitis: A Systematic Review
Source: Nutrients. 2025 Feb 21;17(5):762. doi: 10.3390/nu17050762 (PMC11901544; doi:10.3390/nu17050762)
Supplement: Supplementary file 1 [file nutrients-17-00762-s001.zip › nutrients-3477352-supplementary.pdf]

nutrients MDPI

Supplementary Materials:

Table S1. Indicators for database searches.

**Article-screening strategy**	KEYWORDS: "A":herbal medicine", "B": plant extracts", "C":"essentials oils, "D":phytochemicals, "E":anti-inflammatory, "F":anti-inflammatory activity, "G":dentistry, "H":oral health, "I"periodontal therapy, "J":gingivitis, "K":periodontitis.<br>Boolean Indicators: (("A" OR "B" OR "C" OR "D") AND ("E" OR "F") AND ("G" OR "H" OR "I" OR "J" OR "K")).<br>Timespan: 18 December 2024 to 24 December 2024.<br>Electronic databases: PubMed; Scopus; Web of Science.

Table S2. Featured research in the qualitative analysis and their characteristics.

Authors (Year)	Study Design	Aim	Material and methods	Outcomes
T S Hrishi et al., 2016 [147]	Randomized controlled clinical trial	To evaluate the efficacy of a ***camellia sinensis*** -based dentifrice as an adjunct to scaling and root planing (SRP) in managing mild to moderate PD.	30 patients with mild to moderate PD divided in 2 groups (Test group: ***camellia sinensis*** - based dentifrice, control group: commercial toothpaste). Clinical parameters: Gingival Index (GI), PI, probing pocket depth (PPD), and clinical attachment level (CAL). Biochemical markers: Antioxidant activity in gingival crevicular fluid (GCF).	Both groups showed clinical improvement due to SRP. The ***camellia sinensis*** dentifrice group had significantly: Greater reductions in gingival inflammation and bleeding. Enhanced antioxidant activity in GCF.
Saman Mahyari et al., 2016 [68]	Randomized, double-blind, placebo-controlled trial	To evaluate the efficacy of a polyherbal mouthwash containing extracts of *Zingiber* officinale,	60 patients with gingivitis divided into three groups (polyherbal, CHX, placebo). Participants used the assigned	Both the polyherbal and CHX groups showed significant improvements in gingival and plaque indices,

		Rosmarinus officinalis, and *Calendula officinalis* (5% w/v) in treating gingivitis, compared to CHX and placebo.	mouthwash twice daily for 14 days. Gingivitis and plaque indices: moodified Gingival Index (MGI), Gingival Bleeding Index (GBI), Modified Quigley-Hein Index (MQH) were assessed at baseline, day 7, and day 14.	with no significant difference between the two treatments. The polyherbal mouthwash was safe and well-tolerated.
Kanyawat Rattanasuwan et al., 2016 [153]	Clinical trial	To evaluate the clinical effect of locally delivered thermo-sensitive ***camellia sinensis*** gel as an adjunct to SRP in chronic PD subjects over a 6-month period.	48 patients with chronic PD, were divided into a test group and a control group. Both groups received standard scaling and SRP. The outcomes assessed included probing PPD, CAL, GI, BOP, and full mouth plaque score (FMPS).	No significant long-term differences were observed between the two groups at the 6-month follow-up.
Mohammad Rayyan et al., 2018 [165]	RCT	To evaluate the effectiveness of a 2% mucoadhesive *grape* seed extract (*GSE)* gel in treating chronic PD.	86 periodontal sites from 5 systemically healthy patients, which were divided into a control group (38 sites) and a *GSE* group (48 sites). Both groups received SRP, and gels were applied over a 9-day period.	Results showed significant improvements in GI and PI for the *GSE* group compared to the control group, but no significant differences were found in PoD.
Alefiya Shabbir Mamajiwala et al., 2018 [169]	Randomized, placebo-controlled clinical trial	To evaluate the effectiveness of CHX and cinnamon extract as ultrasonic coolant agents to reduce bacterial load in dental aerosols.	Participants were divided into three groups: CHX, cinnamon extract, and distilled water (DW) (control).	Both CHX and cinnamon extract significantly reduced bacterial contamination in aerosols compared to DW. Cinnamon extract was shown to be a cost-effective, safe alternative to CHX, with comparable efficacy in reducing bacterial load.

Jinfeng He et al., 2019 [158]	Randomized double-blind, placebo-controlled clinical trial	To evaluate the efficacy and safety of toothpaste containing *Rhizoma Chuanxiong* and Rhizoma Imperatae extracts in reducing gingivitis.	120 participants were randomly assigned to the test group (60 participants) or the control group (60 participants). Clinical assessments of plaque, gingivitis, and bleeding were performed at baseline and at 4, 8, and 12 weeks.	Both the test and control groups showed a decrease in gingival inflammation and bleeding, with significant differences observed in GI, Bleeding Index (BI), and BOP% scores at the end of 12 weeks. The test group showed better improvement compared to the control group.
Scilla Sparabombe et al., 2019 [157]	Prospective, RCT	To evaluate the efficacy and safety of a poly-erbal completely natural mouthwash in patients with moderate to severe PD.	In 2 groups, test group, using the natural mouthwash and control group using a placebo mouthwash, Full Mouth Bleeding Score (FMBS), FMPS, Probing Depth (PD), and Clinical Attachment Loss (CAL) were assessed at baseline and after 3 months.	The natural mouthwash was more effective than the placebo in improving periodontal health.
Jaber Yaghini et al., 2019 [171]	Double-blind, placebo-controlled clinical trial	To evaluate and compare the effects of *aloe vera*-***camellia sinensis***, matrica chamomille, and CHX mouthwashes on gingival inflammatory indices and dental stain index in patients with plaque-induced gingivitis.	Participants were randomly assigned to four groups: *aloe vera*-*camellia sinensis*, matrica, CHX, and placebo. PI, GI, BOP, and dental stain index were measured before and after two weeks. Data were analyzed using SPSS and statistical tests.	*Aloe vera*-***camellia sinensis*** and CHX were equally effective in reducing plaque, gingival inflammation, and BOP. Matrica was less effective and caused more staining. The placebo group showed minimal changes.
Li Cheng et al. 2019 [159]	Clinical trial	To evaluate the effectiveness of *Pudilan* extract-containing toothpaste in controlling dental plaque and	120 participants divided into two groups: an experimental group (Pudilan extract toothpaste) and a control group	The experimental group showed greater and sustained improvement in PI, GI, BI, and BOP% than the control

		improving chronic gingivitis.	(placebo toothpaste). Clinical measurements including GI, PI, BI, and BOP%. *Pudilan* extract containing several active ingredients like flavonoids, alkaloids, and organic acids.	group at 8 and 12 weeks. The control group's progress plateaued. No significant adverse effects were reported, with good taste and tolerance.
Hendrik Jünger et al., 2020 [172]	RCT, double-blind clinical study	48 dentate subjects, mean age 77.5 ± 7.3 years. Randomized into two groups: sage-containing mouthwash (test) vs. water/alcohol-based solution (placebo). Rinsing once daily for 30s over 6 weeks.	Reduction in SBI (Sulcus Bleeding Index) and PI in both groups, with no significant differences between test and placebo. No changes observed in xerostomia, stomatitis, or tooth staining. Sage-containing mouthwash showed no superior effect compared to placebo.	The combination of chemical and plant-derived antibacterials provided a safe and effective approach for the treatment of gingivitis and early-stage PD.
Zaira F Kharaeva et al., 2020 [160]	Clinical comparative study	To distinguish the clinical effects and mechanisms of sodium monofluorophosphate combined with xylitol and herbal extracts from Swiss medicinal plants (Chamomilla recutita, Arnica montana, Echinacea purpurea, and Salvia officinalis) in managing gingivitis and the initial stage of PD.	50 patients with gingivitis and early-stage PD, were divided into a control group using toothpaste with 1450 ppm sodium monofluorophosphate and xylitol, and an experimental group using the same chemical components plus herbal extracts (Chamomilla recutita, Arnica montana, Echinacea purpurea, and Salvia officinalis).	The herbal extracts inhibited bacterial defenses against oxidative stress, which was not observed with the CTP alone. The combination of chemical and plant-derived antibacterials provided a safe and effective approach for the treatment of gingivitis and early-stage PD.
Eltay et al., 2021 [170]	Randomized, controlled, double-blind clinical trial	Evaluated as adjunct to nonsurgical	34 patients with chronic gingivitis randomized 1:1 for 4 weeks.	No adverse reactions reported. *Punica granatum* peel extract

		periodontal therapy.	Intervention: pulsating jet irrigator with 5% *punica granatum* peel extract solution. Control: placebo.	showed potential as an effective alternative for managing chronic gingivitis within the study's limits.
Aslan Kh. Sheregov et al., 2021 [174]	Clinical trial	To study the effectiveness of SFPG in complex therapy for moderate chronic PD developed as an orthodontic treatment complication.	50 patients (18 men, 32 women) with moderate PD; divided into experimental and control groups.	Significant reduction in inflammation, normalization of cytokine levels, and decreased nitrite/nitrate content.
Jin-Young Park et al., 2021 [175]	Multi-centered, randomized, controlled clinical trial	To evaluate the effects of the test treatment on gingival inflammation, inflammatory markers, and patient-reported outcomes (PROMs)	104 patients with gingivitis or Test group: daily intake of 194 mg PMEC capsule for 8 weeks. Control group: placebo. Clinical and immunological parameters assessed at baseline, 4, and 8 weeks.incipient PD.	Significant reduction in MGI in the test group at 4 and 8 weeks. Reduced IL-6 in gingival fluid and increased salivary MMP-9 in the test group. Improved PROMs in the test group compared to placebo.
Yu-Rin Kim et al., 2022 [176]	Randomized, double-blind, placebo-controlled study	To examine the efficacy of a natural mouthwash based on Sambucus williamsii var. koreana in the treatment of PD.	64 patients randomized into two groups: saline solution gargle (control) vs. Sambucus williamsii var. coreana extract gargle (test).	Significant improvement in O'Leary index, PI, GI, and subgingival plaques in the test group after Treatment and After 5 Days ($p < 0.05$). Antibacterial effect observed against both gram-positive and gram-negative bacteria.
Bharani Krishna Takkella et al., [58]	Double-blinded, RCT	To evaluate the anti-inflammatory properties of phytocompounds from *Punica granatum* and *Lawsonia inermis* as potential natural alternatives to	60 patients divided into three groups (20 each): *Punica* mouthwash, *Lawsonia* mouthwash and DW (control). Mouthwash used for two weeks.	Significant reduction in salivary AST, ALT, and LDH levels in both *Punica* and *Lawsonia* groups, indicating reduced inflammation. *Punica* was more

		traditional mouthwashes for the treatment of PD,	Salivary enzymes (AST, ALT, LDH) measured before and after using mouthwash. Statistical analysis with SPSS 22.	effective than *Lawsonia*. DW group showed increased enzyme activity, indicating disease progression.
Jae-Suk Jung et al., 2024 [173]	Multicentered, randomized, double-blind, placebo-controlled trial	To evaluate the effectiveness of a PMEC compound in reducing inflammatory biomarkers and promoting oral health in patients with moderate to severe gingivitis,	104 subjects with gingivitis or incipient PD. Intervention: PMEC vs. placebo for 8 weeks. Primary outcome: inflammatory biomarkers from GCF. Secondary outcome: clinical parameters.	Clinical parameters improved in both groups, but no significant intergroup differences

Table S3. It presents a tabular summary of the Risk of Bias assessment for 17 studies, evaluated across six domains.

AUTHORS AND YEARS	D1	D2	D3	D4	D5	D6	Overall
Hrshi, T.S et al. (2016)	+	+	+	+	+	+	+
Mahyari, S.et al. (2016)	+	+	+	+	+	+	+
Rattanasuwan,K. et al. (2016)	+	+	+	+	-	+	+
Rayyan,M. et al. (2018)	+	+	+	+	-	+	+
Mamajiwala,A.S et al (2018)	+	+	-	+	+	+	+
He,J. et al.(2019)	+	+	+	+	+	+	+
Sparabombe,S. et al. (2019)	+	+	+	+	+	+	+
Yaghini,J. et al. (2019)	+	+	+	+	-	+	+
Cheng,L. et al. (2019)	+	+	+	+	+	+	+
Jüngeret,H. al. (2020)	+	+	+	+	+	+	+
Kharaeva,Z.F. et al. (2020)	+	+	+	+	+	+	+
Eltay,E.G. et al. (2021)	+	+	+	+	+	+	+

Sheregov,A.Kh. et al. (2021)	+	+	+	+	+	+	+
Park,J-Y.et al. (2021)	+	+	+	+	+	+	+
Kim,Y-R. et al. (2022)	+	+	+	+	+	+	+
Takkella,B.K. et al. (2023)	+	+	-	+	-	+	-
Jung,J.S. et al. (2024)	+	+	+	+	+	+	+

**Domains:**	**Judgement:**	
D1: Bias due to confounding.	Very Hight	!
D2: Bias arising from the measurement of the exposure.	Hight	X
D3: Bias in the selection of participants in the study (or into the analysis).	Some Concerns	-
D4: Bias due to post-exposure interventions.	Low	+
D5: Bias due to missing data.	No Information	?
D6: Bias arising from measurement of the outcome.		

Table S4. Plant Species and Their Bioactive Components with Beneficial Effects on Oral Health.

Plant	Bioactive Components	Beneficial Effects	Reference Studies
Camellia sinensis (Green Tea)	Catechins, flavonoids, polyphenols	Anti-inflammatory, antimicrobial, antioxidant	Hrishi et al. (2016), Rattanasuwan et al. (2016)
***Zingiber* officinale** (Ginger)	Gingerols, shogaols	Anti-inflammatory, antimicrobial	Mahyari et al. (2016)
Rosmarinus officinalis (Rosemary)	Rosmarinic acid, flavonoids	Antioxidant, antimicrobial	Mahyari et al. (2016)
Calendula officinalis (Calendula)	Triterpenoids, flavonoids	Anti-inflammatory, antimicrobial	Mahyari et al. (2016)
Propolis	Flavonoids, phenolic acids	Antibacterial, antioxidant, immunomodulatory	Sparabombe et al. (2019), Yaghini et al. (2019)
Salvia officinalis (Sage)	Carnosic acid, rosmarinic acid	Anti-inflammatory, antimicrobial	Sparabombe et al. (2019), Jünger et al. (2020)
Rhizoma Chuanxiong	Alkaloids, flavonoids	Anti-inflammatory, circulatory stimulant	He (2019)
Rhizoma Imperatae	Saponins, flavonoids	Antioxidant, antimicrobial	He (2019)
Punica granatum (Pomegranate)	Punicalagin, tannins, polyphenols	Antibacterial, anti-inflammatory	Eltay et al. (2021), Takkella et al. (2021)
Lawsonia inermis (Henna)	Lawsone, flavonoids	Antioxidant, antimicrobial	Takkella et al. (2021)
Aloe vera	Anthraquinones, polysaccharides	Anti-inflammatory, soothing	Yaghini et al. (2019)

Plant	Bioactive Components	Beneficial Effects	Reference Studies
Garcinia mangostana (Mangosteen)	Xanthones, flavonoids	Anti-inflammatory, antimicrobial, antioxidant	Jung et al. (2024), Park et al. (2021)
**Sambucus williamsii var. coreana**	Flavonoids, phenolic acids	Antibacterial, inflammation reduction	Kim et al. (2022)

Table S5. Summary of Plant-Based Products and Their Effects on Oral Cavity.

Plant (Scientific Name)	Family	Type of Preparation	Main Bioactive Compounds	Actions in the Oral Cavity	Mechanism of Action
Camellia sinensis (Green Tea)	Theaceae	Extract, mouthwash, gel	Catechins (EGCG), flavonoids, polyphenols	Antioxidant, anti-inflammatory, antimicrobial	Reduces oxidative stress, inhibits bacterial adhesion, modulates inflammatory response
Punica granatum (Pomegranate)	Lythraceae	Peel extract, mouthwash	Punicalagins, flavonoids, ellagic acid	Antibacterial, anti-inflammatory, collagen synthesis promoter	Inhibits MMPs, reduces IL-1β and PGE2, promotes tissue repair
Lawsonia inermis (Henna)	Lythraceae	Leaf extract, mouthwash	2-hydroxynaphthoquinone, xanthones, flavonoids	Antimicrobial, anti-inflammatory, analgesic	Inhibits bacterial growth, modulates cytokine release
Rosmarinus officinalis (Rosemary)	Lamiaceae	Essential oil, extract	Carnosic acid, rosmarinic acid, flavonoids	Antioxidant, anti-inflammatory, antibacterial	Scavenges free radicals, inhibits inflammatory pathways
Zingiber officinale (Ginger)	Zingiberaceae	Extract, essential oil	Gingerol, shogaol, paradol	Antimicrobial, anti-inflammatory	Reduces pro-inflammatory cytokines, inhibits bacterial proliferation
Calendula officinalis (Marigold)	Asteraceae	Extract, gel	Faradiol monoester, flavonoids, triterpenoids	Anti-inflammatory, healing agent	Inhibits COX-2, promotes wound healing

Plant (Scientific Name)	Family	Type of Preparation	Main Bioactive Compounds	Actions in the Oral Cavity	Mechanism of Action
Propolis	Various sources	Standardized extract, mouthwash	Flavonoids, phenolic acids, terpenoids	Antimicrobial, anti-inflammatory, antioxidant	Inhibits bacterial biofilm formation, modulates immune response
Aloe vera	Asphodelaceae	Gel, mouthwash	Acemannan, anthraquinones, polysaccharides	Anti-inflammatory, wound healing, antimicrobial	Stimulates fibroblast activity, reduces inflammatory mediators
Cinnamomum verum (Cinnamon)	Lauraceae	Essential oil, extract	Cinnamaldehyde, eugenol, polyphenols	Antibacterial, antifungal, anti-inflammatory	Disrupts bacterial membranes, inhibits inflammatory pathways
